# Implementation of an Extended-Infusion Piperacillin-Tazobactam Dosing Protocol: Unexpected Findings when Monitoring Safety and Compliance with Smart Pump Technology

**DOI:** 10.3390/pharmacy7040169

**Published:** 2019-12-11

**Authors:** Nathaniel J. Rhodes, Jenna Lopez, Cecilia K. Pham, Helga Brake, Michael Fotis, Spencer E. Harpe, Sean Avedissian, Marc H. Scheetz

**Affiliations:** 1 Department of Pharmacy Practice, Chicago College of Pharmacy, Midwestern University, Downers Grove, IL 60515, USA; sharpe@midwestern.edu (S.E.H.); mschee@midwestern.edu (M.H.S.); 2 Pharmacometrics Center of Excellence, Midwestern University, Downers Grove, IL 60515, USA; 3 Department of Pharmacy, Northwestern Memorial Hospital, Chicago, IL 60611, USA; 4 Department of Pharmacy, Loyola University Medical Center, Maywood, IL 60153, USA; jlopez65@midwestern.edu; 5 Department of Pharmacy, Jesse Brown Veteran Affairs Medical Center, Chicago, IL 60007, USA; 6 Department of Pharmacy, Rite Aid Pharmacy #5910, Benicia, CA 94510, USA; cpham47@midwestern.edu; 7 The Institute for Innovations in Care and Quality, Illinois Hospital Association, Naperville, IL 60563, USA; hbrake@ihastaff.org; 8 Feinberg School of Medicine, Medical Education, Northwestern University, Chicago, IL 60611, USA; michael.fotis@northwestern.edu; 9 Antiviral Pharmacology Laboratory, University of Nebraska Medical Center (UNMC) Center for Drug Discovery, UNMC, Omaha, NE 68198, USA; snausc@gmail.com; 10 College of Pharmacy, University of Nebraska Medical Center, Omaha, NE 68198, USA

**Keywords:** antimicrobial stewardship, extended-infusion, piperacillin-tazobactam, smart pump

## Abstract

Compliance with recommended infusion rates was evaluated before, during, and after the implementation of extended-infusion (EI) piperacillin-tazobactam at an academic medical center. Software-controlled infusion-pump alert data were studied for piperacillin-tazobactam administrations before and after implementation of a four-hour EI protocol. Compliance was analyzed 16 weeks before (pre-EI), two weeks after (peri-EI), and an additional 16 weeks after (post-EI) protocol implementation. We defined potential harm as a programmed infusion rate exceeding the recommended rate, possible harm as a programmed infusion aborted by the user, and compliance as reversion to recommended rates. Potential and possible harm were standardized to 1000 patient days. Overall, 3110 alerts were identified during the period. Potential harm per 1000 patient days for pre-, peri-, and post-EI were 0, 6.12, and 1.05 (*p* < 0.001). Possible harm per 1000 patient days for the pre-, peri-, and post-EI were 0.33, 21.9, and 5.02 (*p* < 0.001). Compliance after an initial potential harm alert occurred more often post-EI (0.4 per 1000 patient days vs. 0 per 1000 patient days for pre- and peri-EI; *p* < 0.001), while alerts remaining in non-compliance were more prevalent if they initially occurred during the peri- and post-EI vs. pre-EI (6.1 and 0.6 per 1000 patient days vs. 0 per 1000 patient days; *p* < 0.001) period. Piperacillin-tazobactam infusions were administered faster than recommended during implementation (i.e., peri-EI) despite standardized orders.

## 1. Introduction

Beta-lactams are among the most frequently utilized antimicrobials among hospitalized patients [1]. Piperacillin-tazobactam has been utilized heavily in many healthcare institutions for empiric and directed treatment of serious bacterial infections. Increasing Gram-negative resistance to beta-lactams, including piperacillin-tazobactam, has led to the increased adoption of extended-infusion (EI) and continuous-infusion (CI) dosing schemes to improve the percent of the dosing interval that free (*f*) drug concentrations remain above the minimum inhibitory concentration (MIC), or %*f*T_>MIC_. 

While the benefits of administering beta-lactams like piperacillin-tazobactam by EI and CI have been documented in infection models [2,3,4] as well as a growing number of clinical studies [5,6,7,8,9,10,11,12,13,14], less is known about the technical challenges encountered that can impact EI or CI protocol compliance after implementation [15,16,17,18]. Previous studies have shown that protocol non-compliance rates may be as high as 10% after implementation of an EI protocol, but these analyses were limited to the post-protocol (post-EI) period and relied on direct observations of infusions, which may be subject to measurement biases (i.e., Hawthorne effects) during implementation [15,19]. 

Computer-linked, software-controlled, infusion pump technology (smart pumps) can aggregate infusion details (programmed infusion rates, programmed volumes, and durations) into electronic databases [20]. These databases capture records of end-user and smart pump interactions and are less likely to introduce bias. Further, these smart pumps can be used to identify unplanned infusion schemes such as unplanned rapid infusion rates. For beta-lactams, rapid infusions have the potential to decrease %*f*T_>MIC_ by allowing drug concentrations to drop below the MIC prematurely. These unanticipated and unplanned effects can potentially lead to patient harm [21]. The purpose of this study is to describe the infusion schemes used before and after implementation of an EI-dosing protocol at our institution using smart pump infusion data as a surveillance method.

## 2. Materials and Methods

### 2.1. Study Design 

We conducted a single center, retrospective, before and after study at Northwestern Memorial Hospital over an 8-month period surrounding the implementation of an EI-dosing protocol for piperacillin-tazobactam. We generated a report containing all piperacillin-tazobactam infusion alerts recorded with smart pumps (Alaris®, CareFusion, San Diego, CA, USA). Evaluable alerts were generated between May 1, 2011, and December 31, 2011. Alerts generated in the operating rooms or the emergency department were excluded from the analysis as EI-dosing was not implemented in these care areas. The alert database captured all end-user (e.g., nurse) actions related to the infusion. After Pharmacy and Therapeutics Committee review and approval, the EI-dosing protocol was implemented on August 24, 2011, after establishing information technology support, electronic ordering capacity, and the dissemination of informational and educational materials. Evaluable piperacillin-tazobactam infusions included 4.5 g, 3.375 g, and 2.25 g doses prior to implementation of the EI-dosing protocol. Only 4.5 g and 3.375 g doses were evaluable after implementation of the EI-dosing protocol as other doses were phased out post-implementation. The smart pump database was queried for piperacillin-tazobactam infusions during the study period. Information collected included the pump identification number, the patient location, the origination and disposition date and time for the alert, the programmed volume of the infusion, the programmed infusion rate, and the programmed piperacillin-tazobactam dose (i.e., 2.25 g, 3.375 g or 4.5 g). The alert was classified as possible harm or potential harm with an alert disposition of compliance or non-compliance (see Section 2.2). Financial databases were queried for the monthly number of inpatient admission days (i.e., patient days) during the study period to allow standardization of error rates to 1000 patient days. This study was determined to be exempt by the Institutional Review Boards at both Northwestern University (Chicago, IL, USA) and Midwestern University (Downers Grove, IL, USA).

### 2.2. Intervention

Prior to implementation of the EI-dosing protocol, physicians, nurses, and pharmacists were educated on the rationale for and logistics related to the EI-dosing protocol. Educational and support materials were disseminated throughout the institution and posted to the local intranet. The educational and support materials outlined the institutionally approved renal dosing guidelines (based on creatinine clearance), co-infusion (i.e., Y-site) compatibilities, and the contact information for the antimicrobial stewardship program pharmacist on call to assist with troubleshooting. The following time periods were defined for the analysis: 16 weeks before implementation of the EI-dosing protocol (pre-EI), 2 weeks after implementation of the EI-dosing protocol (peri-EI), and 16 weeks after the peri-EI period (post-EI). The institution recommended infusion rate for piperacillin-tazobactam during the pre-EI period was 100–200 mL/hr per 50–100 mL prepared bag (corresponding to a 30-min. infusion time) and was 25 mL/hr per 100 mL bag (corresponding to a 4-h infusion time) in the peri- and post-EI periods, respectively as shown in Table 1. The stewardship pharmacist coordinated implementation of the EI-dosing protocol with information technology specialists and served as a resource throughout the peri- and post-EI periods. 

Inputting an infusion rate in excess of the recommended infusion rates during the pre- and peri-EI period resulted in a notification to the end-user (e.g., nurse) of a rate-limit violation. Initially in the pre-EI period, the end-user could override the warning and proceed with an incorrect infusion rate. After multiple instances of overly rapid programmed infusion rates were identified in the peri-EI period, the Antimicrobial Stewardship Program in collaboration with the Pharmacy Informatics group removed the ability of the end-user to override the recommended infusion rate limits and forced the end-user to comply with the recommended infusion rates or to cancel the infusion. In the post-EI period, cancellations were therefore classified as non-compliance.

### 2.3. Definitions

For the purposes of this study, the unit of analysis was considered as the alert rate. Specifically, alerts were generated in response to administration attempts by the smart pump interface, wherein one or more possible infusion attempts could have been made for a given patient (i.e., one or more alerts representing harm could have occurred to the same patient for the same infusion) though patient level data were not available. Potential harm was defined as a programmed infusion rate in excess of 200 mL/hr during the pre-EI period and a programmed infusion rate in excess of 25 mL/hr during the peri- and post-EI periods. This definition for potential harm was based on PK studies that have shown the different pharmacodynamics when using different infusion strategies [12,22]. Possible harm was defined as an attempt by the end-user to voluntarily abort the initially selected pre-programmed infusion because the ultimate disposition of the infusion remained unclear. In addition to possible harms, protocol compliance was also assessed. Resolution of the alert with an action that would be deemed compliant with the protocol was defined as alteration of the programmed infusion rates to revert to the recommended infusion rate limits for each period. Terminal non-compliance rates per 1000 patient days were calculated for the pre-, peri- and post-EI periods, respectively. At the beginning of the pre-EI period, the end-user could override the warning and proceed with an incorrect infusion rate. After multiple instances of overly rapid programmed infusion rates were identified, the Antimicrobial Stewardship Program in collaboration with Pharmacy Informatics removed the ability of the end-user to override the recommended infusion rates limits and forced the end-user to comply with the recommended infusion rates or else cancel the infusion. In the post-EI period, cancellations were therefore classified as non-compliance.

### 2.4. Statistical Analysis

Error rates for potential harm and possible harm and compliance per 1000 patient days were tabulated across all study periods. Continuous variables were evaluated across groups using one-way ANOVA or Kruskal–Wallis tests, as appropriate. Categorical variables were evaluated using Chi-squared or Fisher’s exact tests, as appropriate. Statistics were calculated using Intercooled Stata version 13.1 (Statacorp, College Station, TX, USA) with the exception that rate-based variables (e.g., number of alerts per 1000 patient days) were evaluated using the Mantel–Haenszel extension of the Chi-squared test (Epi-Info 7, CDC, Atlanta, GA, USA). A threshold level of alpha was set at 0.05 for all comparisons.

## 3. Results

Overall, 3110 piperacillin-tazobactam infusion alerts were generated during the eight-month study period from 504 unique infusion devices. The distribution of alert types included 1591 (51.2%) infusion override attempts, 1049 (33.7%) infusion reprogramming attempts, and 470 (15.1%) infusion cancellations in total. The majority of infusion alerts were associated with the 3.375 g dose of piperacillin-tazobactam compared to the 2.25 and 4.5 g doses (90.3% vs. 0.8 and 8.9%, respectively). The majority of alerts were in general medicine (49.4%), followed by critical care (37.2%). Alerts were less common in hematology/oncology (13.0%), and obstetrics and gynecology (0.4%). Infusion alert generation spiked between the pre- and peri-EI periods then fell in the post-EI period (2.7 vs. 223 vs. 30.5 per 1000 patient days, respectively; *p* < 0.001). 

### 3.1. Programmed Infusion Rates and Administered Infusion Rates

The distribution and characteristics of standardized infusion alerts generated across the study period are shown in Table 2. The median (interquartile range, IQR) programmed infusion rates were significantly higher in the pre-(200 (20–500)) and peri-EI periods (50 (25–200)) compared to the post-EI period (25 (20–50); *p* < 0.001).

### 3.2. Potential Harm and Possible Harm Alerts Over Time

**Figure 1:**
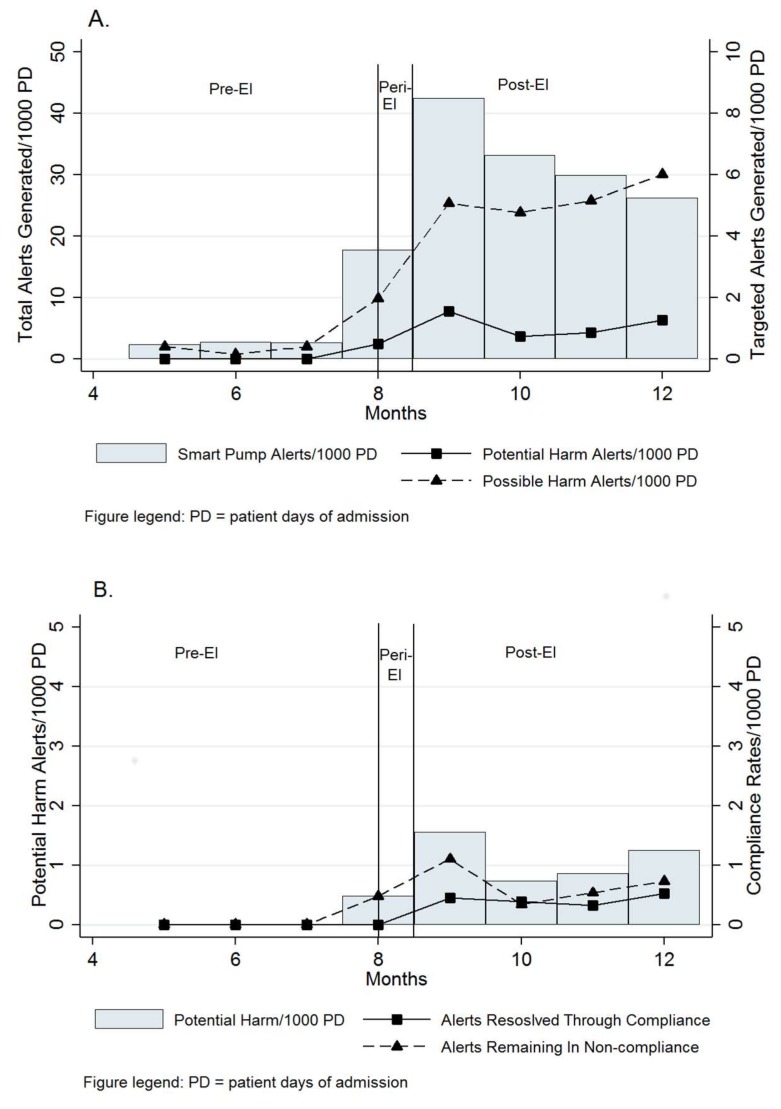
Standardized rates of possible harm and potential harm associated with an EI-dosing protocol implementation. DOT, day of therapy; PD, patient days of occupancy; Pre-EI, days 0 to 115 of the study period; Peri-EI, days 116-128 of the study period; Post-EI, days 129-245 of the study period; possible harm alerts; potential harm alerts.). (**A**) shows the total aggregate alerts generated during the study period across study months and periods. The second y-axis shows the rate (per 1000 patient days) of possible and potential harm alerts across study months and periods. (**B**) shows only the potential harm alerts generated across study months and periods. The second y-axis shows the rate (per 1000 patient days) of potential harm alerts resolved through compliance and the proportion remaining non-compliant across study months and periods. EI-dosing protocol implemented in the eighth month. Bars are aligned with the first y-axis ((A) total alerts generated/1000 PD; (B) potential harm alerts generated/1000 PD). Lines are aligned with the second y-axis ((A) targeted alerts generated/1000 PD; (B) compliance rates/1000 PD). Lines of demarcation separate the peri-EI period from the pre- and post-EI periods in each panel.

The disposition of standardized infusion alerts generated across the study period is shown in Table 2. Alerts representing potential harm and possible harm per 1000 patient days spiked between the pre- and peri-EI periods, as shown in Figure 1A. The average potential harm rates per 1000 patient days rose and fell over the pre-, peri-, and post-EI periods (0 vs. 6.12 vs. 1.05; *p* < 0.001). The average possible harm rates per 1000 patient days also rose and fell over the pre-, peri-, and post-EI periods (0.33 vs. 21.9 vs. 5.02; *p* < 0.001). With respect to dose, alerts representing potential harm were more likely to be associated with piperacillin-tazobactam infusions of 3.375 g compared to 2.25 and 4.5 g doses (*p* = 0.001). However, alerts representing possible harm were not associated with piperacillin-tazobactam dose (*p* = 0.27). With respect to care area, alerts representing potential harm were less likely to occur in general medicine (*p* = 0.018). Alerts representing potential harm were numerically more common in critical care (*p* = 0.076) compared to other areas. Alerts representing possible harm were not associated with any patient care area (*p* = 0.28). Similar to the overall analysis, infusions classified as potential and possible harm rose and then fell during the three study periods within each unit (*p* < 0.001), with the exception of obstetrics and gynecology which remained flat over the three study periods (*p* = 0.37). 

### 3.3. Alerts and Terminal Protocol Non-Compliance

Resolution of potential harm alerts via compliance or non-compliance across the entire study period is shown in Figure 1B. Non-compliance with smart pump rate limits increased across the entire study period. The baseline non-compliance rate during the pre-EI period was 2.33 per 1000 patient days. The non-compliance rate during the peri-EI period increased to 201 per 1000 patient days (*p* < 0.001). Non-compliance post-EI then decreased to 25.5 per 1000 patient days (*p* < 0.001). The rate of non-compliance from peri-EI to post-EI decreased eight-fold. However, it did not return to baseline during the study period. 

Alerts representing potential harm resolved through compliance increased from 0 per 1000 patient days in the pre- and peri-EI periods to 0.42 per 1000 patient days in the post-EI period (*p* < 0.001). Alerts representing potential harm that remained terminally non-compliant were highest in the peri-EI period vs. pre-, and post-EI periods (6.12 vs. 0 and 0.63 per 1000 patient days respectively, *p* < 0.001). 

## 4. Discussion

We found unexpected and concerning levels of protocol deviations during implementation of an EI-dosing protocol for piperacillin-tazobactam at our institution. Specifically, we identified that smart-pump users were programming overly rapid infusions that could have resulted in patient harm. Electronic surveillance allowed for an technology-based intervention to minimize non-compliant infusion practices. In other words, it allowed us to detect practices in need of improvement during peri-EI and intervene. However, our data also demonstrated that some proportion of patients prescribed EI received different infusion rates. Additionally, we were surprised to find that terminal non-compliance in the post-EI period did not return to zero, suggesting a need for ongoing education on EI protocols well after initially rolling them out. Even with active surveillance, new protocols will take time before maximal uptake is achieved and altered processes become the norm. Stewardship programs should be vigilant for such challenges after EI and CI implementation. This is the first report, to our knowledge, to quantify EI-protocol compliance using an electronic database of infusion alerts. Utilization of quality assurance databases to monitor compliance with EI protocol implementation appears to be a novel and useful tool for antimicrobial stewardship. When available, information gleaned from smart pump databases should be reviewed to ensure appropriate protocol compliance. By feeding these data back to the end-user, an impetus can be created to help change outdated or problematic behaviors.

Our findings are generally similar to those of other investigators utilizing more conspicuous methodologies. Xamplas et al. conducted a quasi-experimental study on the implementation of an EI piperacillin-tazobactam dosing protocol in a large academic medical center [15]. The authors piloted the EI-dosing protocol within the medical intensive care unit and subsequently implemented the protocol throughout the adult population of their institution. Following initial education efforts and development of electronic order sets for prescribers, pharmacists prospectively identified orders for EI-doses of piperacillin-tazobactam and actively confirmed that piperacillin-tazobactam was appropriately administered and recorded programmed infusion rates for patients receiving EI-dosing. Appropriate piperacillin-tazobactam administration was also confirmed using the medication administration record. They found that approximately 10% (*n* = 19 instances) of administrations were non-compliant after EI-protocol implementation and 90% (*n* = 17/19) of protocol deviations were related to overly rapid programmed infusion rates. We found that terminal non-compliance with the protocol was highest during the peri-EI period at 6.12 per 1000 patient days and decreased to 0.63 per 1000 patient days in the post-EI period. The most common cause of terminal non-compliance during the peri-EI period was the voluntary abortion of the infusion attempt by the end-user, which remained elevated in the post-EI period. With the methodology described here, we are not able to decipher if these non-compliant events were eventually rectified. Xamplas et al. were unable to compare historic rates of non-compliance with post-intervention period non-compliance. Additionally, the authors relied on non-compliance data gathered while directly observing the end-user, which may have led to possible underestimation of latent non-compliance due to a Hawthorne-like effect. Our analysis removed the observer interaction with the end-user, and may provide a less-biased approximation of non-compliance.

Another study in 2011 by Heinrich et al. looked at the barriers to adherence after the implementation of a piperacillin-tazobactam EI guideline at their academic medical center [16]. They prospectively monitored infusions at the patient bedside to ensure the piperacillin-tazobactam product, dose, rate, and concomitant infusions were correct and compatible. They found an approximate 2% missed dose rate and a 4% delayed dose rate. It is not clear what the rates of non-compliance would have been in a different setting (e.g., not actively monitored by the presence of a practitioner) or what rates were when this level of monitoring was removed (i.e., Hawthorne effect). 

Limitations to our analysis must be considered. First due to the indirect capture of data, our analysis was based on retrospective data and subject to all biases associated with such analyses. Second, the database lacked unique end-user identifiers, thus prohibiting analyses on potential outlier practitioners. However, patient care area stratification data allowed for general feedback and education for certain “hot-spots” of non-compliance. Third, because the dataset was not linked to patient identifiers, we were unable to correlate the alerts in our database with clinical outcomes. Fourth, a limitation of the smart pump software utilized during the study period allowed the end-user to reattempt previously aborted piperacillin-tazobactam infusions by re-entering the infusion generically. Hence, terminal compliance could have been better than the rates reported here because it is unlikely that all cancelled EI infusions were entered as generic infusions subsequently, but we were unable to capture this. Our results demonstrate the worst-case scenario. 

## 5. Conclusions

Using an inconspicuous method for classification of infusion strategies, this study identified non-compliant infusion rates for piperacillin-tazobactam. Infusion rates were significantly higher peri-implementation of an EI-dosing protocol and were subsequently decreased with a targeted intervention. However, a portion of infusions remained outside of guidelines up to three months after protocol implementation. Hospitals and antimicrobial stewardship programs seeking less biased feedback and information upon which to target education and interventions may wish to analyze available smart pump infusion-alert databases.

## Figures and Tables

**Table 1:** Extended-infusion dosing protocol and recommendations at Northwestern Memorial.

	Pre-Implementation	Post-iImplementation
Available doses (in g)	2.25/3.375/4.5	3.375/4.5
Dosing interval used (in h)	4 to 12	8 to 12
Infusion duration (in h)	0.5	4
Recommended infusion rates (in mL/h)	200	25
Locations where non-EI doses permitted	All	ED/ORs

Abbreviations: ED, emergency department; ORs, operating rooms

**Table 2:** Distribution and resolution on infusion alerts generated across study periods.

	Pre-EI	Peri-EI	Post-EI	*p-*Value
Number of alerts	*n* = 216	*n* = 509	*n* = 2385	─
Number of inpatient admission days	81,111	2288	78,273	─
Duration of time in period (days)	115	13	117	─
Standardized alert rate, per 1000 patient days	2.7	223	30.5	<0.001
Programmed infusion rates, median (IQR)	200 (20–500)	50 (25–200)	25 (20–50)	<0.001
Fold prolonged/excess rate, median (IQR)	1 (0.1–3)	2 (0.4–8)	1 (0.8–2)	<0.001
Patient care areas contributing any alerts				0.07
Critical care/emergency, n/N (%)	118 (54.6)	136 (26.7)	903 (37.9)	0.17
Medical/surgical, n/N (%)	73 (33.8)	317 (62.3)	1147 (48.1)	0.90
Hematology/oncology, n/N (%)	25 (11.6)	55 (10.8)	323 (13.5)	0.13
Labor/delivery, n/N (%)	0 (0)	1 (0.2)	12 (0.5)	0.17
Potential harm alert rate, per 1000 patient days	0	6.12	1.05	<0.001
Potential harm rate resolving in compliance, per 1000 patient days	0	0	0.42	<0.001
Potential harm rate remaining in non-compliance, per 1000 patient days	0	6.12	0.63	<0.001
Possible harm alert rate, per 1000 patient days	0.33	21.9	5.02	<0.001

Abbreviations: IQR, interquartile range; EI, extended-infusion; Pre-EI, days 0–115 of the study period; Peri-EI, days 116–128 of the study period; Post-EI, days 129–245 of the study period
